# Duodenal Diverticulosis as an Unusual Cause of Severe Abdominal Pain

**DOI:** 10.7759/cureus.10586

**Published:** 2020-09-22

**Authors:** Wajd A Aljabri, Mohammed Hasosah, Abeer AlMehdar, Dohaa Bakhsh, Faris O Alturkistany

**Affiliations:** 1 Pediatric Gastroenterology, King Saud Bin Abdulaziz University for Health Sciences, National Guard Hospital, Jeddah, SAU; 2 Pediatric Gastroenterology, King Saud Bin Abdulaziz University for Health Sciences, King Abdullah International Medical Research Center, Jeddah, SAU; 3 Pediatric Radiology, King Saud Bin Abdulaziz University for Health Sciences, King Abdullah International Medical Research Center, Jeddah, SAU; 4 Medicine, King Saud Bin Abdulaziz University for Health Sciences, National Guard Hospital, Jeddah, SAU

**Keywords:** duodenal diverticulosis, diverticulitis, abdominal pain, child

## Abstract

Duodenal diverticulosis (DD) is a rare disease in children, and its etiology is unknown. Here, we report a 13-year-old boy with severe abdominal pain. A diagnosis of DD was made based on clinical and image findings. He responds to analgesic, antibiotic and nutritional treatment. The early identification of child with DD as potential cause of severe abdominal pain with pancreatitis is important - because delayed diagnosis might lead to irreversible consequences - to avoid morbidity and mortality, and unnecessary surgery.

## Introduction

The duodenum is considered the second most common site of diverticula with the colon being the commonest site [1]. The prevalence of duodenal diverticulosis (DD) is estimated to be 23%; however, it is dependent on the different modalities used in diagnosis [2]. DD can be the cause of a congenital or an acquired phenomenon. Furthermore, they can come in two forms either intramural or extramural [1-4]. The most common manifestation would be acquired and extramural [1]. The majority are asymptomatic with less than 10% of the patients having nonspecific symptoms such as abdominal pain [1-2]. Serious complications of DD might include diverticulitis, which is a very rare condition in the pediatric age group [3]. Many various imaging modalities are used to detect such abnormality. CT is the modality of choice for DD regardless the age of presentation. This paper reports a rare case of duodenal diverticulitis in a 13-year-old boy who presented with severe abdominal pain, vomiting, and fever. Review of the literature is also provided in the discussion.

## Case presentation

**Figure 1:**
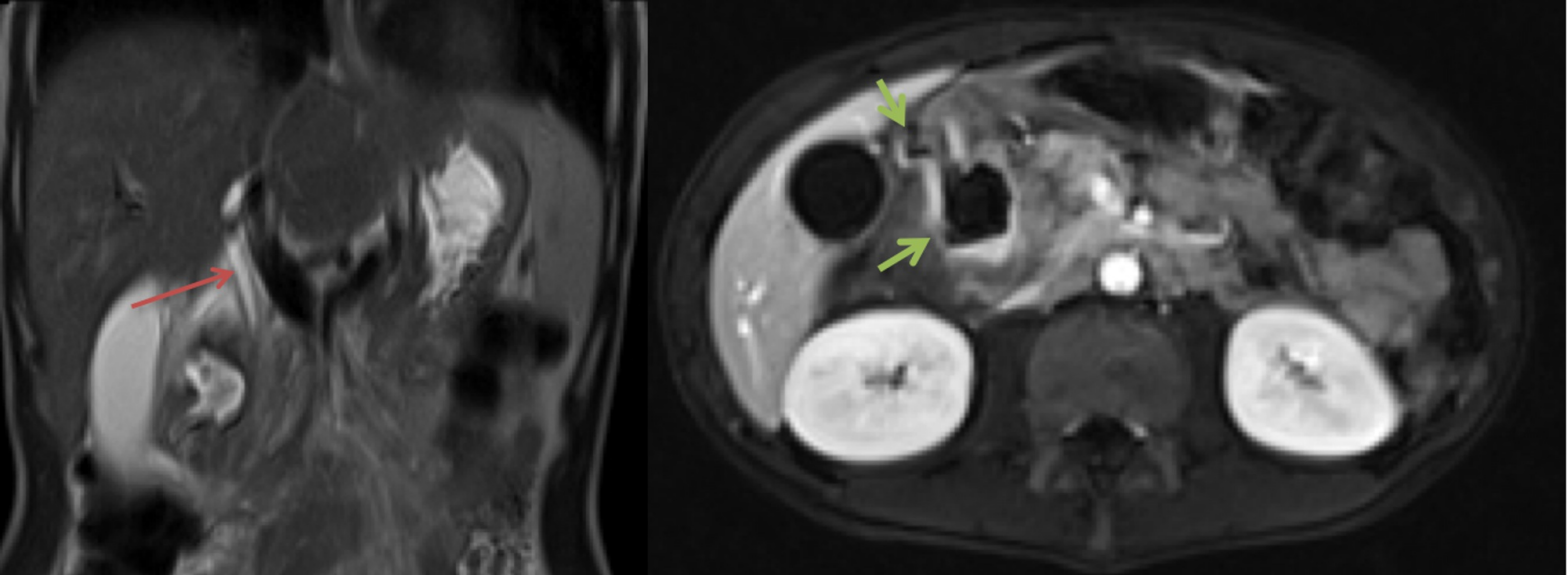
MRI selected images: Coronal T2WI (a) and axial T1WI (b) with fat sat and post IV contrast administration demonstrate the duodenal diverticula (green arrows) with air fluid level. The MR images confirm the diverticulitis changes and exclude the pancreatic pathologies. Note the distended CBD (red arrow).

A 13-year-old boy presented to the emergency department with severe right upper quadrant pain. It has been increasing in severity for the last three days. The pain was associated with vomiting, and low-grade fever. There was no contact history with ill patient. His medical and surgical history were unremarkable. On abdominal examination, there was periumbilical and epigastric tenderness. A positive Blumberg sign was present. His laboratory results were as following: hemoglobin 13.9 (normal range: 12.2-15.3 gm/dL), white blood cell 7.8 (normal range: 6-16 × 10^9^/L), platelet 412 (normal range: 150-450 × 10^9^/L), serum triglycerides 108 (normal range: < 150 mg/dL), calcium 9.9 (normal range: 8-10.5 mg/dL), serum amylase 560 (normal range: 40-140 U/L) and serum lipase 190 (normal range: 0-160 U/L). Serum alanine transaminase, aspartate aminotransferase, alkaline phosphatase, gamma-glutamyl transferase, albumin, and bilirubin were normal. An abdominal X-ray showed nonspecific bowel gas distribution with no signs of bowel obstruction, and no free air or pneumatosis intestinalis. A broad-spectrum antibiotics and acetaminophen were started and the patient kept nil per mouth. A computed tomography (CT) of the abdomen demonstrated the circumferential thickening in the wall of the gastric antrum and pylorus as well as the duodenum. Periduodenal edema and free fluid was identified with the head of pancreas being swollen and edematous suggestive of pancreatitis. There was slight distention of the common bile duct (CBD) with enhanced wall. Two out-pouches from the second part of the duodenum were identified from the medial and superior walls that is fluid filled and showed air fluid level (Figure 1).

**Figure 2:**
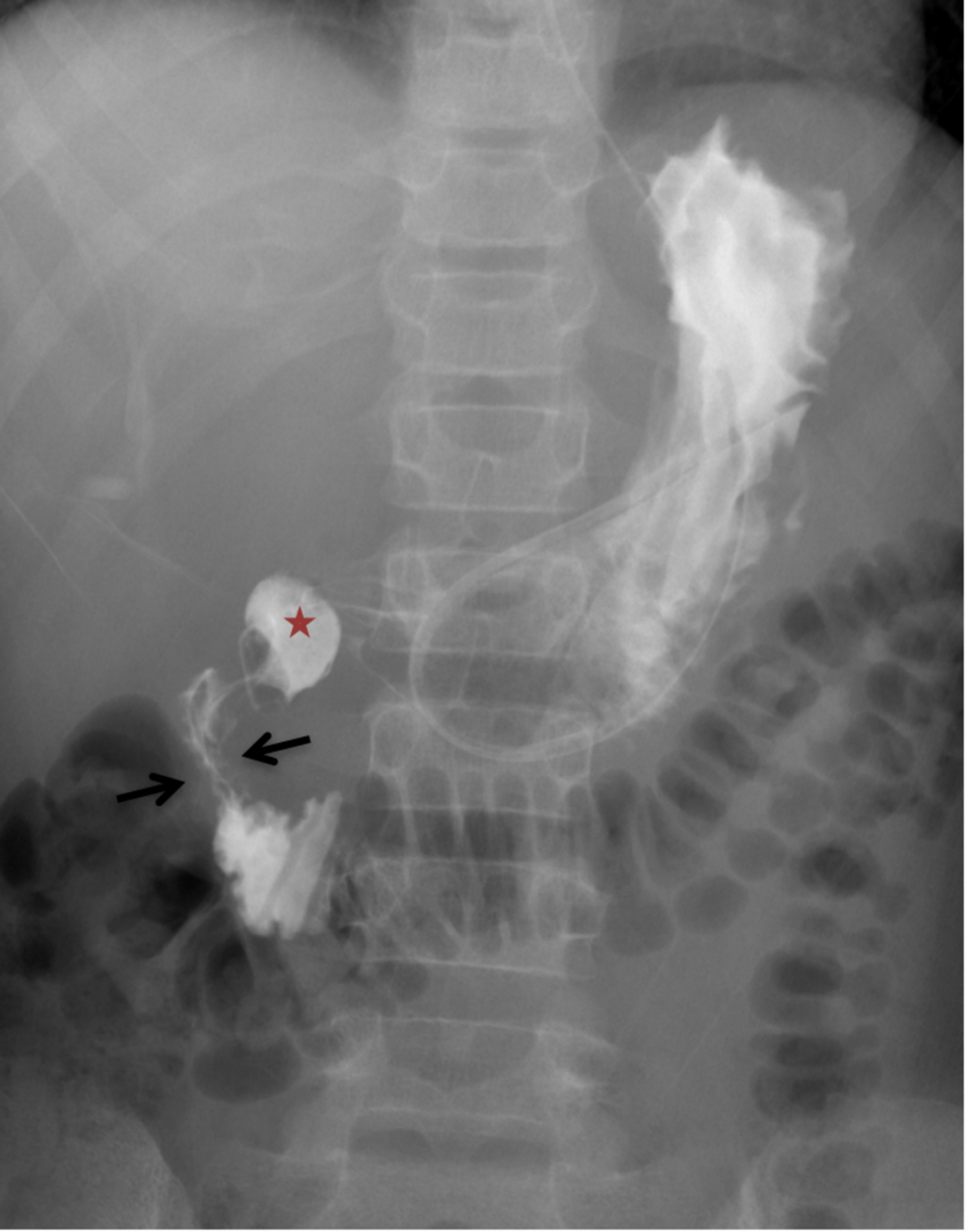
Selected frontal radiograph from gastrografin swallow study demonstrates the contrast filled out-pouch from the second part of the duodenum (star). Note the narrowing of the duodenal lumen with thumb printing (arrows) changes suggestive of bowel wall edema.

These are highly suggestive of diverticuli. Further imaging workup has been performed. The magnetic resonance cholangiopancreatography (MRCP) reported abnormal wall thickening of the antrum, pylorus, and first and second parts of the duodenum. The two large duodenal diverticula were described again in Figure 2.

**Figure 3:**
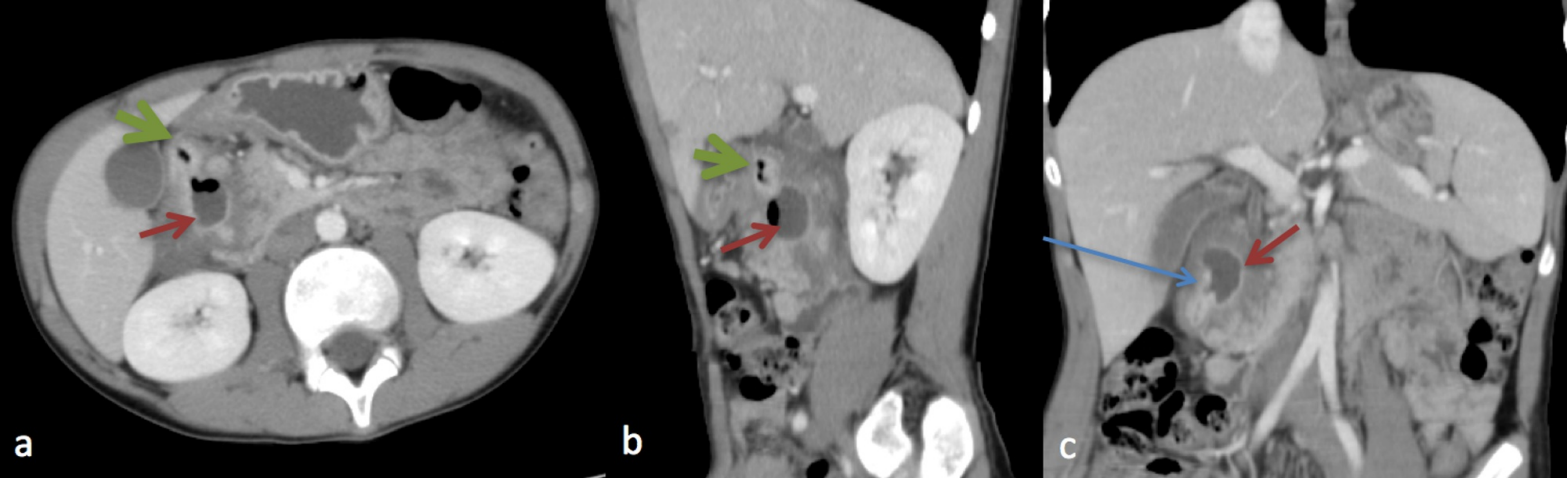
Selected contrast enhanced CT scan of the abdomen in axial (a), with sagittal (b) and coronal (c) reconstructed images, demonstrates the duodenal diverticuli (green and red arrows) outpouching from the second duodenum, medial in location. The larger diverticulum shows air fluid level. The diverticular neck is better depicted in the coronal plane (blue arrow). Noted are the periduodenal inflammatory changes with bowel wall thickening and edema.

The head of pancreas was found to be swollen and inseparable from the duodenum and the diverticulum. Signs of acute pancreatitis and dilated CBD were present. Swallowing study demonstrated the duodenal diverticulum as an out-pouch from the medial wall of the second duodenum that was filled with contrast throughout the exam (Figure 3). It also was noted that the duodenal wall edema with thumb-printing changes is consistent with the diverticulitis and enteritis. Seven days later, esophagogastroduodenoscopy (EGD) revealed erythema in duodenum with multiple ulcerations. It showed a 1-cm diverticulum in the duodenum, confirming a similar finding from images. Histopathological findings showed acute duodenitis with eosinophilic infiltrate in the lamina propria. A diagnosis of DD was made based on the clinical presentation, endoscopy, and radiological findings. The patient was treated with ceftazidime, metronidazole, ketorolac, and total parenteral nutrition (TPN). At the two months follow-up, he was completely asymptomatic, and his pancreatic enzymes remained normal.

## Discussion

The duodenum is considered the second most common site of diverticula following the colon [1]. Depending on the modality used for diagnosis, the prevalence of duodenal diverticula was revealed. Five to 10% on barium radiographic series and 22% on autopsy were reported to have DD [4]. Both sexes are equally predominant of getting DD with age varying from 26-69 years [2]. DD can be recognized as congenital or acquired. It can be further classified as intramural or extramural [4]. The most common manifestations were acquired and extramural DD. DD is mostly distributed in the second or third part of the duodenum, and is commonly located near the ampulla of vater [4,5].

Due to the nature of the disease, 90% are asymptomatic and discovered incidentally during an upper endoscopy or barium study [5]. The remaining 10% present with various symptoms that are nonspecific including abdominal pain, intermittent diarrhea, constipation, weight loss, and fever. These symptoms may appear due to inflammation, hemorrhage, perforation, or obstruction of the biliopancreatic structure [5]. It has been shown that the most common cause of duodenal perforation is duodenal diverticulitis [4]. The clinical presentation of complicated duodenal diverticulosis can mimic several gastrointestinal diseases such as peptic ulcer disease, acute cholecystitis, acute pancreatitis, or appendicitis [2].

Previous reports suggest that radiographic studies do not routinely add to the accuracy of preoperative diagnosis of diverticular disease [2]. However, it can be of use in cases of pneumoperitoneum if complicated with perforation. Uncomplicated duodenal diverticula can be single or multiple [6]. On barium studies, diverticula manifest as contrast filled saccular outpouchings of the duodenal lumen [7]. The ultrasound appearance has been described as a persistent bright linear or concave echo that obscures visualization of the normal pancreatic head [6]. Abdominal cross sectional imaging has been reported to be a useful diagnostic tool in defining duodenal pathology and overcoming the difficulties of clinical and ultrasound examinations. CT is the modality of choice for diverticulitis with a high sensitivity and specificity [8]. The CT appearance of a duodenal diverticulum includes a saccular outpouching, which may resemble a mass-like structure interposed between the duodenum and the pancreas that contains air, an air-fluid level, fluid, contrast material, or debris [2]. When filled with fluid, periampullary diverticula may mimic a pancreatic or choledochal cyst [7]. The CT features of duodenal diverticulitis may include wall thickening and stranding of the surrounding soft tissues and adjacent mesenteric or retroperitoneal fat. Surrounding extraperitoneal free air is not rare; however, pneumoperitoneum is rare [2].The use of orally administered contrast material and IV-administered contrast material may have been helpful in defining the anatomy and demonstrating an inflamed duodenal diverticulum [2]. Coronal reformatted images seem to demonstrate the diverticular neck to a better extent than the axial images. This is in accordance with the anatomical location of most of the duodenal diverticula in the mesenteric border of the bowel loop [9]. In magnetic resonance (MR) imaging, the diverticula are more frequently identified by gas, a gas-fluid/contrast level, or debris in the diverticulum, adjacent to the normal duodenal lumen. The use of negative oral contrast for magnetic resonance cholangiopancreatography (MRCP) has been suggested [6]. MRCP is very helpful to exclude biliary and pancreatic pathologies and complications. Endoscopic images, colonoscopy, and barium enema are avoided until the acute manifestations are resolved. Those modalities can be used to establish the extent of the disease and to rule out other differential diagnosis [10].

Patients with asymptomatic duodenal diverticulosis do not require treatment. The management in symptomatic patients is based on their clinical presentation. Patients who present with diarrhea and malabsorption due to small intestinal bacterial overgrowth are treated with antibiotic therapy such as rifaximin [11]. Small bowel obstruction due to enterolith impaction of the diverticula is either treated with enterotomy and stone extraction or manual crushing and milking of the stone distally into the colon [12]. In other patients who present with choledocholithiasis due to juxtapapillary diverticula, management can be successfully done by the use of endoscopic retrograde cholangiopancreatography with sphincterotomy and stone removal followed by laparoscopic cholecystectomy [13]. Following a restricted diet and an antibiotic therapy are the mainstay of treatment in clinically stable patients with uncomplicated diverticulitis [1, 11-16]. Diverticulitis can be complicated by refractory gastrointestinal bleeding or infections [14]. Other complications may include duodenal fistula, intra-abdominal abscesses, sepsis, and perforation. Surgical management should be limited only to those patients with serious complications [1,14]. These surgical interventions include open or laparoscopic-assisted resection of the involved segment [15,16]. Perforation is one of the most serious yet rare complications of diverticulitis [1]. Although perforated duodenal diverticulitis is known to be treated surgically, several studies have shown that non-operative management should be the initial step for certain cases [17].

## Conclusions

Similar cases of DD in adults can be easily diagnosed and identified using specific imaging protocols and guidelines. This case is reported because of its rare cause of abdominal pain and challenging diagnostic imaging findings in a 13-year-old child. The early identification of child with DD as potential cause of severe abdominal pain with pancreatitis is important. It needs a skilled physician to detect the DD and to order the appropriate imaging techniques. Delay in making the diagnosis of DD due to its rare occurrence in the children population might lead to drastic repercussions that will lead to high morbidity and mortality that can easily be avoidable.
